# Simple low-cost construction and calibration of accurate pneumotachographs for monitoring mechanical ventilation in low-resource settings

**DOI:** 10.3389/fmed.2022.938949

**Published:** 2022-08-01

**Authors:** Ramon Farré, Miguel A. Rodríguez-Lázaro, David Gozal, Gerard Trias, Gorka Solana, Daniel Navajas, Jorge Otero

**Affiliations:** ^1^Unitat de Biofísica i Bioenginyeria, Facultat de Medicina i Ciències de la Salut, Universitat de Barcelona, Barcelona, Spain; ^2^CIBER de Enfermedades Respiratorias, Madrid, Spain; ^3^Institut Investigacions Biomèdiques August Pi Sunyer, Barcelona, Spain; ^4^Department of Child Health, The University of Missouri School of Medicine, Columbia, MO, United States; ^5^Department d'Infrastructures i Enginyeria Biomedica, Hospital Clínic, Barcelona, Spain; ^6^Faculdade de Engenharias e Tecnologias, Universidade Save, Maxixe, Mozambique; ^7^Institute for Bioengineering of Catalonia, Barcelona Institute of Science and Technology, Barcelona, Spain

**Keywords:** tidal volume, flow measurement, mechanical ventilation, calibration, pneumotachograph, low- and middle-income countries

## Abstract

Assessing tidal volume during mechanical ventilation is critical to improving gas exchange while avoiding ventilator-induced lung injury. Conventional flow and volume measurements are usually carried out by built-in pneumotachographs in the ventilator or by stand-alone flowmeters. Such flow/volume measurement devices are expensive and thus usually unaffordable in low-resource settings. Here, we aimed to design and test low-cost and technically-simple calibration and assembly pneumotachographs. The proposed pneumotachographs are made by manual perforation of a plate with a domestic drill. Their pressure-volume relationship is characterized by a quadratic equation with parameters that can be tailored by the number and diameter of the perforations. We show that the calibration parameters of the pneumotachographs can be measured through two maneuvers with a conventional resuscitation bag and by assessing the maneuver volumes with a cheap and straightforward water displacement setting. We assessed the performance of the simplified low-cost pneumotachographs to measure flow/volume during mechanical ventilation as carried out under typical conditions in low-resource settings, i.e., lacking gold standard expensive devices. Under realistic mechanical ventilation settings (pressure- and volume-control; 200–600 mL), inspiratory tidal volume was accurately measured (errors of 2.1% on average and <4% in the worst case). In conclusion, a simple, low-cost procedure facilitates the construction of affordable and accurate pneumotachographs for monitoring mechanical ventilation in low- and middle-income countries.

## Introduction

The importance of mechanical ventilation in patients requiring intensive care for acute respiratory failure has been recently underlined as a public health issue worldwide in the context of the COVID-19 pandemic. This major healthcare emergency has highlighted the need for sufficient mechanical ventilators to be readily available even in countries with the most developed health care systems (1). However, despite the acute need for mechanical ventilators because of COVID-19 or other future pandemics, the lack of these fundamental intensive care medical devices is an important chronic problem in low- and middle-income countries (LMICs) (2). Indeed, given the very high cost of conventional commercially available ventilators, these medical devices are essentially unaffordable with the low resources available in LMICs at present and in the foreseeable future. Therefore, having a sufficient number of ventilators available in low-resource settings requires an effort to develop much more affordable devices (3, 4), a proposal that has received accrued interest in recent years because of the current pandemic (5). Importantly, mechanical ventilators suitable for LMICs should be robust and easy to check and repair locally, since technical servicing and maintenance of medical devices is also a chronic problem in LMICs, as reflected by the fact that medical devices from philanthropic donations tend to become useless within short periods after their installation due to the lack of appropriate maintenance (6). This is particularly relevant in the case of mechanical ventilators for ICUs since the accuracy of these devices is more likely to be compromised particularly in developing countries (7).

Measuring inspiratory airflow and computing the associated tidal volume is crucial for achieving suitable gas exchange levels while providing protective ventilation (8) thereby avoiding ventilator-induced lung injury (9) and patient-self-inflicted lung injury (10). Measurement of flow and volume, which is particularly susceptible to errors in mechanical ventilators (11, 12), is usually carried out by built-in conventional pneumotachographs with a flow-sensing resistor based on a mesh screen (Lilly-type) or on parallel capillaries (Fleisch-type). Such pneumotachographs have the important advantage of being linear (pressure drop across the resistor proportional to flow). However, their use in mechanical ventilators for LMICs is fraught with two major drawbacks resulting from the very small size of their sensing components (mesh screens or capillaries). Specifically, their fabrication is complex and thus expensive, and their correct function can be challenged in case of poor maintenance usually resulting in partial obstruction of the sensing capillaries or mesh screen orifices. Other potential technical approaches for flow measurement involve exceedingly expensive devices (e.g., ultrasound-based sensors or hot-wire anemometers) with potentially lower robustness (13).

In this context, the work described herein aimed to devise and test a procedure for very easily constructing and calibrating low-cost pneumotachographs that are robust, accurate, and affordable for users in low-resource regions. The devised procedure, which does not require expensive gold standard measuring devices (14, 15), was tested for monitoring flow and volume during mechanical ventilation.

## Methods

### Construction of the low-cost pneumotachograph

**Figure 1:**
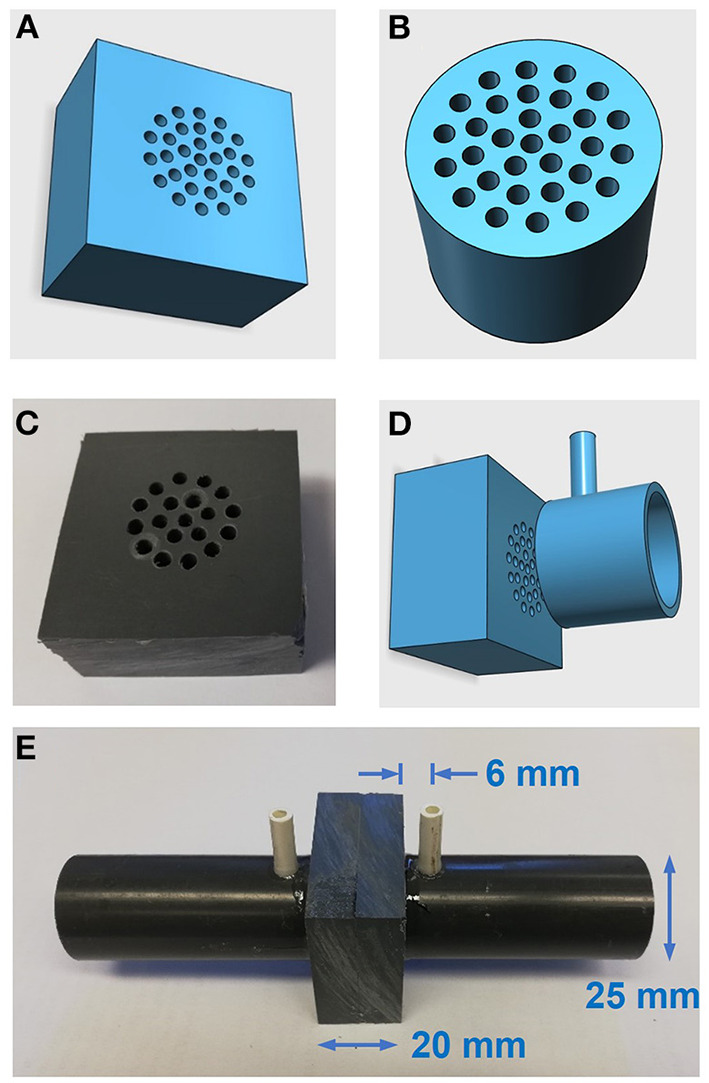
Low-cost pneumotachograph. **(A,B)** Diagrams of resistors for the pneumotahographs. **(C)** Photograph of the manually-drilled resistor. **(D)** Diagram of resistor and (one side) standard PVC tube to assemble the pneumotach. **(E)** Photograph of the assembled pneumotachograph.

The simple and low-cost procedure proposed for constructing an accurate pneumotachograph is based on creating a resistor by drilling a series of parallel narrow cylindrical channels, as illustrated in Figure 1. We used conventional drill bits of 2.0, 2.5, or 3.0 mm in diameter with a domestic Dremel-type drill. Resistors were made by drilling polyvinyl chloride (PVC) material, either cylinders (2.5 cm in diameter, 2.0 cm in length) or 2 cm-thick rectangular pieces. In both cases, the pneumotachograph was built by assembling the resistor with inlet-outlet tubes having connectors for a differential pressure transducer to sense the resistor pressure drop caused by the airflow to be measured (Figure 1). To guide the drilling and tube attachment positions, the pattern was first drawn on paper and then transferred to the PVC piece. For a general non-linear resistor, the relationship between airflow (V′) and differential pressure signal (P) is described by the classical Rohrer model (16): $P\;=\;K_{1}V^{\prime }\;+\;K_{2}V^{{}^{\prime }2}$. Accordingly, effective resistance R (R = P/V') is R = $K_{1}\;+\;K_{2}V^{\prime },\;$and hence K_1_ and K_2_ represent the linear and non-linear components of the resistor, respectively.

### Simple calibration of the pneumotachograph from two maneuvers of known volume

**Figure 2:**
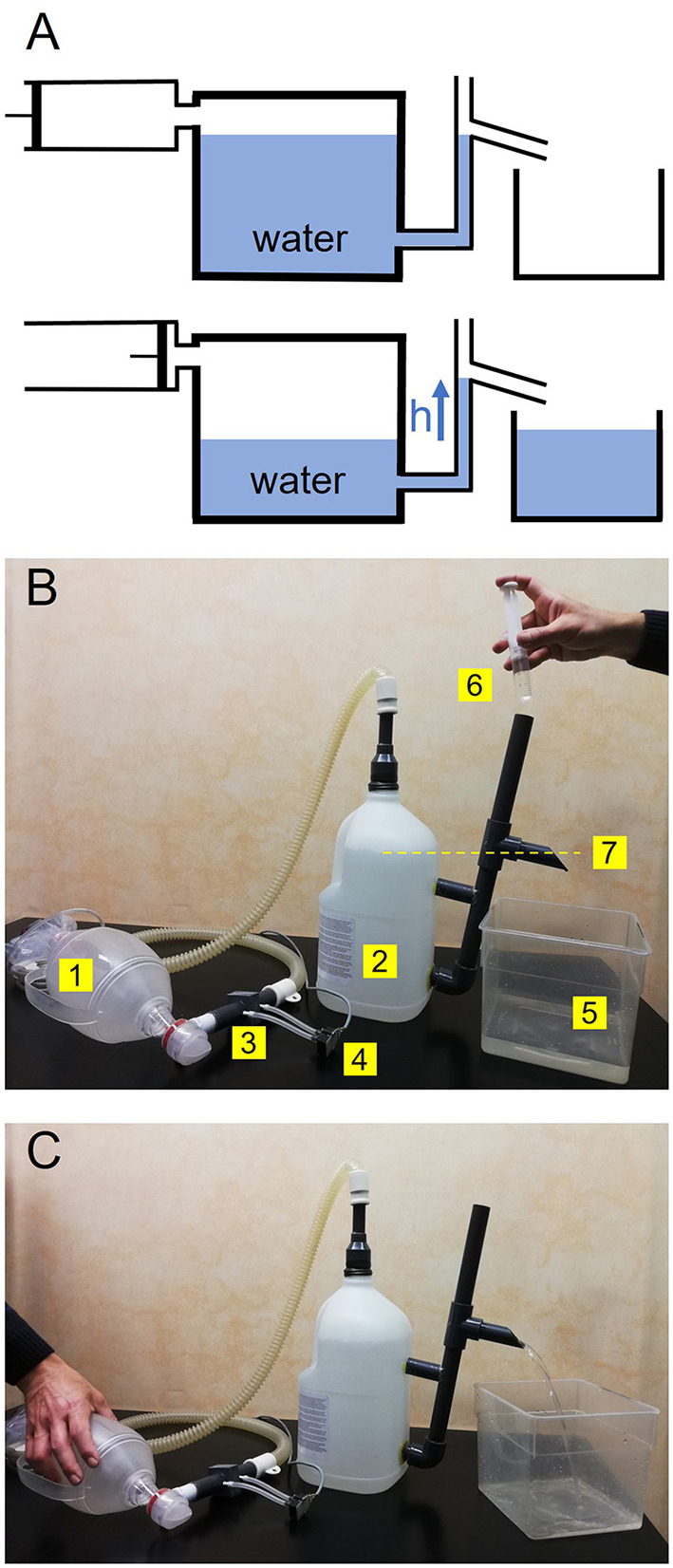
**(A)** Water displacement-based method for measuring the air volume generated by a syringe. See tet for explanation. **(B)** Experimental setting implemented for calibrating a pneumotachograph from two-maneuver volumes assessed by water displacement. The air when compressing a resuscitation bag (1) is introduced into the chamber (2) through the pneumotachograph to be calibrated (3) connected to a differential pressure transducer (4). The water to be displaced from the chamber is collected by a recipient (5). Before starting the maneuver, the chamber is carefully filled with water (6) until achieving the level of the outlet (7), as indicated by the yellow line. **(C)** During the maneuver, an operator is compressing the resuscitation bag and the displaced water is collected by the recipient to measure the total maneuver volume. The pneumotachograph calibration parameters (K_1_ and K_2_) are computed by combining the pressure signals recorded and the volumes in two different maneuvers (Supplementary material).

We have devised a simple procedure for accurately calibrating a non-linear pneumotachograph. Indeed, as delineated in greater detail in Supplementary material, K_1_ and K_2_ can be determined from the pressure signals (P_1_ and P_2_) recorded from the pneumotachograph during any two different maneuvers involving known volumes (V_1_ and V_2_, respectively). The proposed setting, which is based on measuring the volume of air from the volume of water displaced by the airflow maneuver, is described in Figure 2A, in which the top and bottom panels correspond to the initial and final times of the maneuver, respectively. The air volume to be measured is introduced into a chamber containing water and displaces the same volume of water outside the chamber which is collected by an external recipient and measured (Supplementary material presents the method development and discussion from the relevant physical laws). Following this approach, we calibrated one of the constructed pneumotachographs from two maneuvers manually carried out with a conventional disposable resuscitation bag (SPUR II adult, Ambu A/S, Ballerup, Denmark) and we measured the maneuver volume by water displacement. Implementation of the setting in Figure 2A was based on a 5-L (16 cm in diameter) common-use plastic bottle and is shown in Figures 2B,C.

We assessed the practical reproducibility of the procedure for calibrating the low-cost pneumotachograph number 2 in Table 1 [connected to a differential pressure transducer (±2 cmH_2_O, LCVR; Celesco, Canoga Park, CA, USA)] from the volumes of the manual maneuver determined by water displacement. To cover a range of flows representative of those commonly used in mechanical ventilation, the maneuvers should include relatively both low and high flows within the clinical ranges. To this end, the resuscitation bag operator (who was blind to any pressure or flow signal) was simply asked to perform two maneuvers: (a) slowly, with an almost constant compression rate to empty the resuscitation bag (≈1 L) in 6–10 s (to target flows in the range 0.1–0.2 L/s), and (b) a fast maneuver to almost empty the bag in 2–3 s to ensure higher flows. As this maneuver offered more possible options to select the rate of bag compression, the operator was asked to carry out four maneuvers of this type.

**Table 1:** K_1_ and K_2_ of the constructed pneumotachographs.

**Pneumotachograph number**	**Diameter (d) and number (N) of** **drilled channels**	**Flow direction**	**K**_1_ **cmH**_2_**O**·**s/L**	**K**_2_ **cmH**_2_**O**·**(s/L)**^2^
1	d = 2 mm, N = 31	Exhalation	0.383	1.323
		Inhalation	0.343	1.284
2	d = 2.5 mm, N =19	Exhalation	0.273	1.115
		Inhalation	0.273	1.232
3	d = 3 mm, N = 19	Exhalation	0.133	0.585
		Inhalation	0.120	0.564
4	d = 2.5 mm, N = 32	Exhalation	0.129	0.389
		Inhalation	0.125	0.370
5	d = 2.75 mm, N = 29	Exhalation	0.083	0.297
		Inhalation	0.089	0.317

*Pneumotachograph's resistors were made by perforating a cylindrical (1, 3, and 4) or a rectangular (2 and 5) piece, as illustrated in Figure 1*.

### Assessment of the low-cost pneumotachograph to monitor mechanical ventilation

To test the actual performance of the simple and low-cost pneumotachograph and its calibration procedures, we tested them when measuring flow and volume during typical mechanical ventilation (Servo 900C, Siemens, Munich, Germany). The pneumotachograph calibrated by the resuscitation bag maneuvers (number 2 in Table 1) was placed in the inspiratory line of the mechanical ventilation tubing. Ventilation was applied to a patient model (Adult SmartLung; IMT Analytics, Buchs, Switzerland) with a respiratory resistance (20 cmH_2_O) and a compliance (20 mL/cmH_2_O) mimicking a patient with severe lung disease. To have the highest possible accuracy reference, a Fleisch-type pneumotachograph (number 2, Metabo, Epalinges, Switzerland) with a pressure transducer (±2 cmH_2_O, LCVR; Celesco, Canoga Park, CA, USA) was also included in series in the inspiratory line of the ventilator. The flow signal V' from the pneumotachograph under test was computed from its K_1_ and K_2_ obtained by water displacement from manual maneuvers and the pressure signal P recorded from the pneumotachograph. The flow signals from both pneumotachographs were recorded and the associated volumes were computed by flow integration. Comparisons were made for different mechanical ventilation settings (for both pressure and volume control modes) covering a realistic range of tidal volumes in patients (200–600 mL). These inspiratory maneuvers covered the ample range of flow magnitudes (up to >1.2 L/s) and waveforms in routine mechanical ventilation in patients with different diseases (Supplementary Figure S2).

## Results

### Construction of simple pneumotachographs

**Figure 3:**
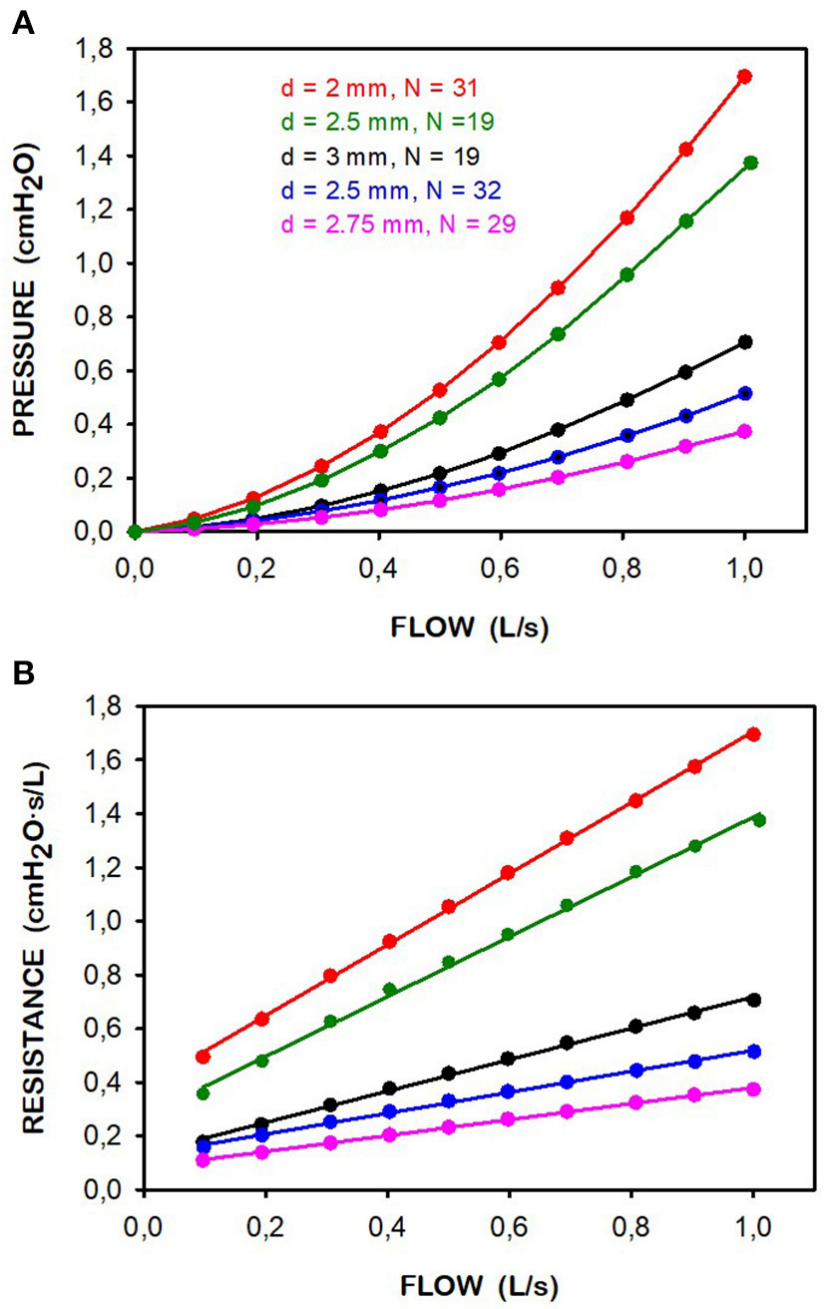
Reference syringe-calibration of the 5 pneumotachographs constructed. **(A)** Relationship between pressure (P) an exhalation flow (V'). **(B)** Corresponding resistance (R) computed as R = P/V'. Points are measured data and lines correspond to fitting a Rohrer model (R = K_1_ + K_2_ · V'). In all cases the quality of the linear fitting was excellent (*r*^2^ >0.9979). The computed K_1_ and K_2_ values are shown in Table 1. Data corresponding to pneumotachographs in Table 1 are in different colors. The legend indicates the diameter (d) and number (N) of drilled channels.

As expected, the P–V' relationship of the low-cost pneumotachographs constructed (Figure 1) was accurately described by the Rohrer model. Figure 3 (top panel) shows the parabolic P–V' relationship measured using a reference calibration syringe (Supplementary material), and Figure 3 (bottom panel) shows the corresponding values of resistance R and the Rohrer model fitting. Table 1 describes the details of the five constructed pneumotachographs and their values of K_1_ and K_2_ for both inhalation and exhalation flow directions.

### Procedure for calibrating the non-linear pneumotachograph from two maneuvers of known volumes

**Figure 4:**
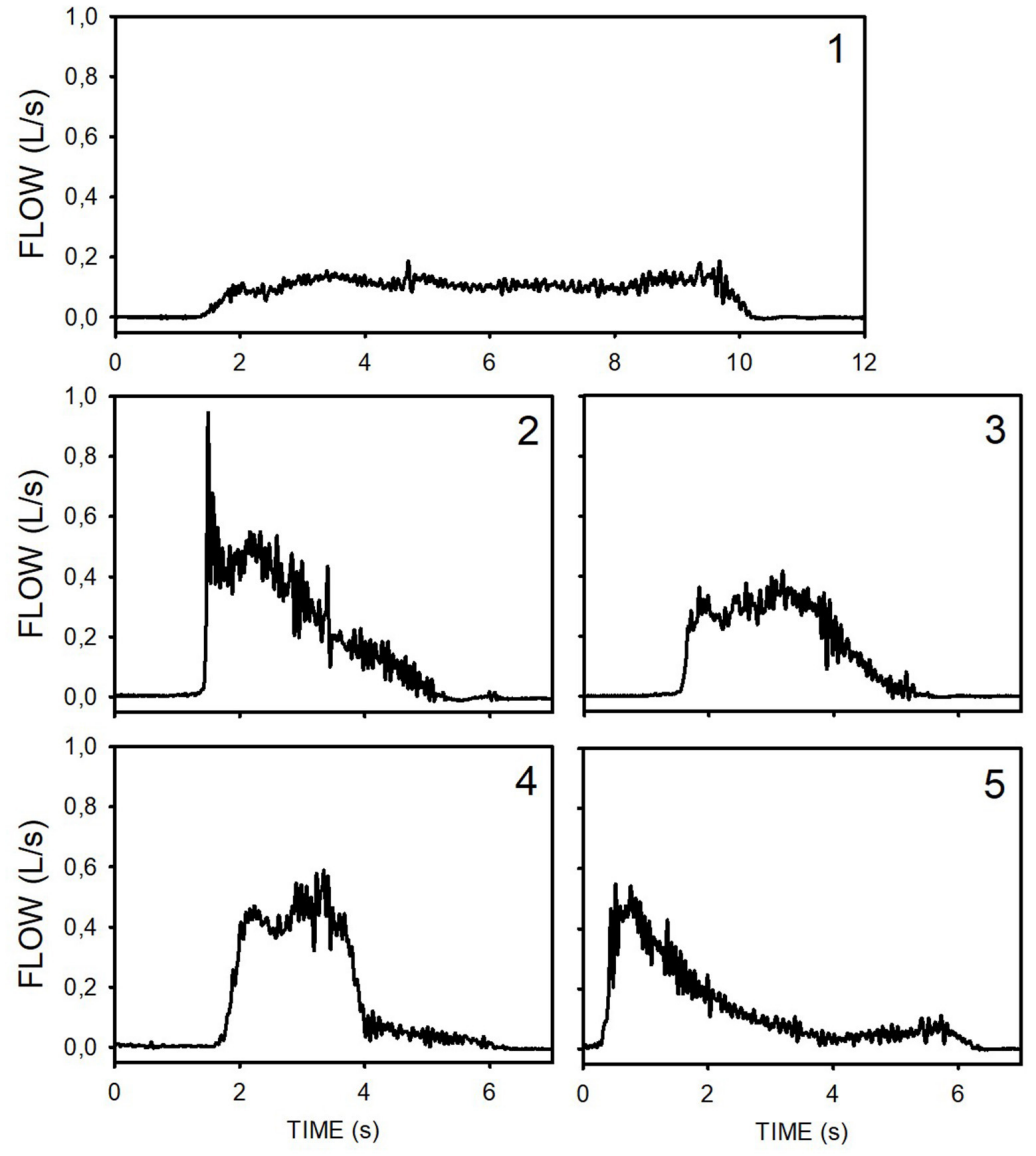
Time course of flow along the four maneuvers carried out manually with the resuscitation bag to calibrate the low-cost pneumotachograph. These flow signals were measured with a reference pneumotachograph. The number of maneuver (1–5) corresponds to those in Table 2.

Figure 4 shows the signals recorded from the resuscitation bag maneuvers, as shown by the flow measured by the reference pneumotachograph. As devised, maneuver 1 had more duration and lower flow than the other four ones which involved higher flows. Table 2 shows the calibration parameters K_1_ and K_2_ of the low-cost pneumotachograph (number 2 in Table 1) determined from the pressure signals recorded during maneuvers 1 and 2 (Figure 4), and using the volumes measured by water displacement. Table 2 also shows the K_1_ and K_2_ values that were computed when the low-flow maneuver 1 was combined with the other high-flow maneuvers, showing minimal differences (<3% coefficients of variation, Table 2) and thus high procedure reproducibility.

**Table 2:** K_1_ and K_2_ of the low-cost pneumotachograph when measured from two manual maneuvers (Table 1).

	**K**_1_ **cmH**_2_**O**·**s/L**	**K**_2_ **cmH**_2_**O**·**(s/L)**^2^
Maneuvers 1 and 2	0.217	1.101
Maneuvers 1 and 3	0.209	1.115
Maneuvers 1 and 4	0.220	1.069
Maneuvers 1 and 5	0.215	1.119
Mean	0.215	1.101
SD	0.005	0.022
CV (%)	2.7	2.1

*Maneuvers 1, 2, 3, 4, and 5 are described in the text and shown in Figure 4*.

### Assessment of the low-cost pneumotachograph and calibration procedure to monitor mechanical ventilation

**Figure 5:**
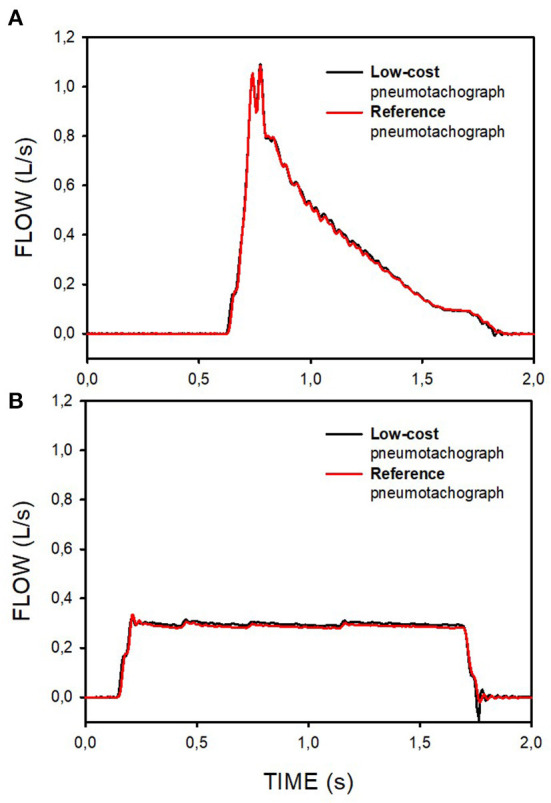
Examples of the flow signals during pressure-controlled **(A)** and volume-controlled **(B)** mechanical ventilation simultaneously measured by a reference pneumotachograph and by the pneumotachograph constructed and calibrated by the low-cost procedures.

The low-cost pneumotachograph with its K_1_ and K_2_ determined by the water-displacement procedure from two resuscitation bag-generated maneuvers (1 and 2 in Figure 4; Table 2) was able to very closely reproduce the flow recorded with the reference Fleisch-type pneumotachograph, as shown by the example of the mechanical ventilation inspiratory flow simultaneously measured by both pneumotachographs (Figure 5).

**Figure 6:**
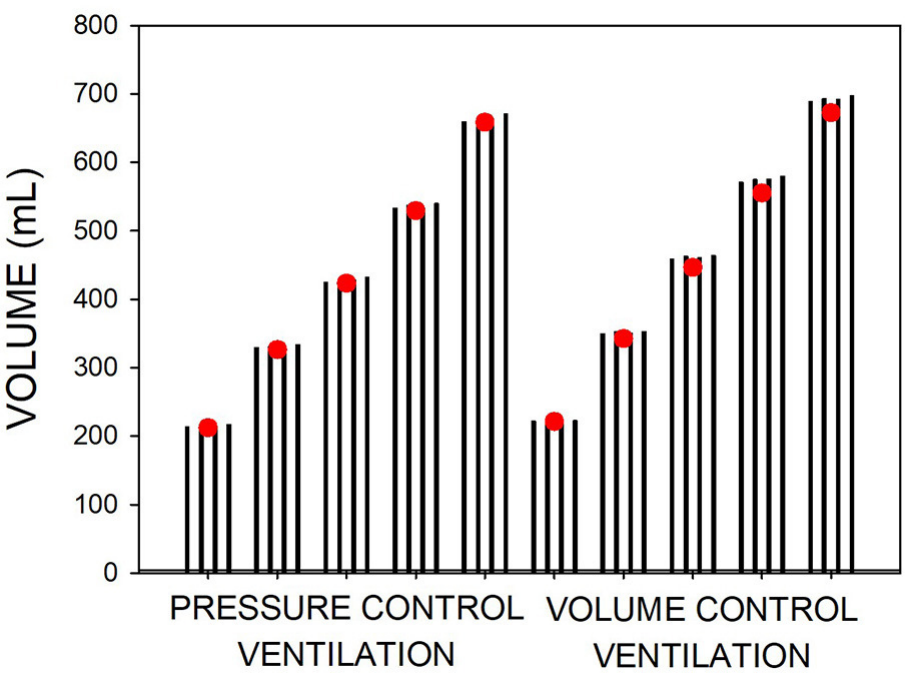
Volume measured during different magnitudes of pressure-control and volume-control mechanical ventilation of a patient model. Volume was simultaneously measured with the low-cost and a reference pneumotachograph (red circles). Each set of four bars corresponds to the volumes measured by the low-cost pneumotachograph when using the different calibration parameters (K_1_, K_2_) obtained by combining different resuscitation bag maneuvers (Table 2).

Figure 6 compares the inspiratory tidal volume measured by the reference pneumotachograph and by the pneumotachograph under test. In this case, the figure depicts the values obtained when using the different K_1_ and K_2_ values derived from different combinations of resuscitation bag maneuvers (Table 2), showing very close concordance. Errors in tidal volume assessment were 2.1% on average and <4% in the worst case.

## Discussion

This study provides a novel approach for simply constructing and calibrating accurate pneumotachographs for measuring flow and volume to be used during mechanical ventilation. The proposed procedure, which does not need laboratory gold standard devices, has been satisfactorily tested showing that a simple manually drill-based pneumotachograph can be calibrated (i.e., K_1_ and K_2_ measured) from maneuvers with a resuscitation bag and water displacement measurement, thereby allowing accurate flow and tidal volume measurements when employed to assess mechanical ventilation. In addition to its use for the training for a correct setting of mechanical ventilation or for experimental studies, the simple pneumotachograph described herein can be incorporated into clinical practice after approval by the competent authorities in each region (local government bodies or hospital ethical committees).

### Pneumotachograph

From a geometrical viewpoint, the type of pneumotachograph proposed and evaluated here can be seen as a modification of the capillary-based approach in conventional Fleisch-type pneumotachographs: capillaries are replaced by parallel cylindrical drilled holes (Figure 1). However, from a flow-dynamics perspective, this geometrical modification implies that the P–V' relationship in the resistor does not follow the linear Poiseuille law, but rather is explained by the quadratic Rohrer model. This model is the description of the P–V' relationship of a perforated-plate model (17), a particular case of porous materials which are typically interpreted with the Darcy-Forchheimer (18) quadratic model in which the resistor is characterized by a linear (K_1_) and a non-linear (K_2_) component conceptually representing the contribution of capillaries and constrictions, respectively (19). In fact, this quadratic model is already well-known in the fields of respiratory physiology and mechanical ventilation, since it was first employed in the seminal work of Rohrer almost one century ago (16) and is nowadays being used to characterize the P–V' relationship in endotracheal tubes (20–22). Figure 3 and Table 1 show that manually drilling different combinations of perforations (in number and diameter) results in pneumotachographs with selectable effective resistances fitting the Rohrer model. Interestingly, a suitable combination, such as in pneumotachograph 3, has a low-flow resistance that is similar to that of conventional pneumotachographs (e.g., 0.2–0.3. cmH_2_O·s/L in Fleisch-II type) presenting maximum resistance well-below 1 cmH_2_O·s/L at peak spontaneous breathing flows (≈1 L/s), enabling its use not only for mechanical ventilation but also for recording spontaneous ventilation in a wide variety of patients and clinical applications such as monitoring resting ventilation or studying sleep apnea.

The K_1_ and K_2_ values in Table 1 show that the pneumotachographs were highly symmetric, suggesting that minor errors would result if both inspiratory and expiratory flows were measured by using the mean values of K_1_ and K_2_ in any given pneumotachograph. Indeed, for flow ranges up to 1 L/s, such error would be very small (<2%) in the pneumotachographs with lower resistance and limited to <4% in the most resistive pneumotachograph. However, this potential error can be easily avoided by using the K_1_ and K_2_ coefficients corresponding to each flow direction. Indeed, inspiratory and expiratory flows/volumes in mechanical ventilation are usually measured by two different pneumotachographs, one placed in the inspiratory line and the other one in the expiratory line of the ventilator circuit. Interestingly, this separated-line setting facilitates the detection (and correction if required) of any potential zero-flow offset in each transducer pressure signals. Such zero-flow correction would avoid any potential error in the flow and volume computation from the pneumotachograph pressure, thereby facilitating the use of low-cost differential pressure transducers even in case they are not thermally compensated (i.e., having slightly variable offset). In this connection, it is worth noting that the current e-market offers differential pressure transducers with excellent performance characteristics (data not shown), requiring no additional conditioning circuit since the pressure output signal is directly provided (e.g., CFSensor, XGZP6897A, ±5 cmH_2_O; ≈11 € in www.Alibaba.com on May 2022). Such ready-to-use transducers connected to the low-cost pneumotachograph allow effective solutions affordable for low resource regions.

Contrary to the complex and expensive fabrication process required by pneumotachographs based on narrow capillaries (Fleisch-type) or mesh screen (Lilly-type), or to alternatives requiring a 3D printer (23), the proposed resistor can be constructed at virtually no cost by simply drilling any material including metals and non-toxic sterilizable polymers (rather than the PVC used in this study as proof of concept). It must be acknowledged, however, that such simplifications in the resistor construction imply the assumption of non-linearity. However, this is not a problem since the P–V' relationship is well-characterized by K_1_ and K_2_, and the computation of flow from pneumotachograph pressure (Equation 2, Supplementary material) is currently straightforward by any processor already incorporated into the simplest mechanical ventilator or even by any stand-alone low-cost Arduino-type based circuit.

The requirement for individual calibration of each resistor is not specific to the proposed pneumotachograph but is in common with conventional Lilly- and Fleisch-type devices. The reason is that the resistor P–V' relationship (either linear or non-linear) critically depends on the exact dimensions of the tiny sensing elements (e.g., diameter in capillary/drills, deviation of exact parallelism in some of the manually drilled holes, size of fiber and holes in a mesh, and rugosity of the surfaces) that cannot be precisely reproduced during the construction and assembling of each pneumotachograph.

### Calibration

A key point in the present study is that the pneumotachograph can be accurately calibrated in a way that does not require a laboratory gold standard device for measuring flow/volume. This approach is based on determining K_1_ and K_2_ from any two maneuvers (for instance manually generated by a resuscitation bag) whose volume is measured by a simple water displacement measurement. The water displacement procedure we implemented by using a common plastic bottle (Figure 2) resulted in very satisfactory results (Figure 6). However, some practical details should be considered and discussed regarding the background hypothesis and the specific experimental setting.

The underlying assumption is that when ambient air (from a syringe or resuscitation bag) is introduced into the chamber (at constant pressure and temperature), the volume of the total air mixture (and thus the volume of displaced water) is the addition of the air volumes before mixing. The fulfillment of this hypothesis depends on the relative humidities of the air volumes before and after mixing. According to the analysis in Supplementary material, the error in estimating the air volume of the maneuver (V_2_) from the difference (ΔV = V_12_ – V_1_) between the volume of the air mixture (V_12_) and the initial air volume in the chamber (V_1_) depends on the partial pressure of water vapor at room temperature, on volumes V_1_ and V_2_ and on the relative humidities of room air and of air in the chamber before and after air mixing. For conditions closely approximating the specific setting we employed (≈20°C, room air at ≈50% humidity, V_1_ ≈ 1 L, V_2_ ≈ 1 L) and reasonably assuming that the air enclosed in the chamber is initially saturated with water vapor (achieved after sufficient time equilibrium with the liquid water), the volume error would be <1.1% (this maximum error value occurring in the case that the mixed air gets 100% water saturation during the very few-seconds of the maneuver). Accordingly, given its negligible value, we did not correct this potential error. However, depending on the specific setting employed to implement Figure 2, easy to apply corrections according to Supplementary material could be required.

Since using a scale is a simple way to assess the volume of displaced water from its weight, we assumed that tap water density is 1 g/L. This provides a sufficiently accurate volume measurement (error <0.5%) since: (a) pure water density at 20°C is 0.9982 g/L and its dependence on laboratory temperature varies by only 0.2% between 15 and 25°C (24), and (b) the densities of mineral or tap water differ from that of pure water by <0.1% (25). Interestingly, we verified that water weight could be measured with sufficient accuracy (±2 mL) using a conventional low-cost kitchen scale. However, assessing the volume of displaced water with a scale can be avoided by simply measuring it directly with a graduated cylinder.

### Practical assessment of the procedure during mechanical ventilation

We tested the proposed procedure when performed under conditions exactly reproducing the ones operationally implemented in clinical settings of LMICs, namely avoiding the use of any laboratory reference device: the pneumotachographs were constructed manually, and they were calibrated by resuscitation bag maneuvers whose volume was measured by water displacement with a setting based on a common-use 5-L plastic bottle. We verified that K_1_ and K_2_ derived from manual resuscitation bag maneuvers carried out by an operator unaware of the aim resulted in very small variation in the calibration coefficients (Table 2), leading to minor variance in tidal volume computation (Figure 6). However, any user of the procedure aiming to ensure more robust K_1_ and K_2_ estimation can repeat the 2-maneuvers process several times to average the resulting parameters (and eventually exclude artifactual data). Using repeated syringe maneuvers was already reported for improving the calibration of conventional pneumotachographs (14, 26).

It is worth mentioning that the errors we found in measuring tidal volume (Figure 6) are similar to the ones reported for mechanical ventilators routinely working in well-serviced ICUs (11), and were minor when compared with the required precision for clinically managing mechanical ventilation. Indeed, the errors in Figure 6 were much lower than the ones accepted for tidal volume measurements. For instance, a recommendation is that volume accuracy is within ± (4.0 mL + 15 % of volume) (27). For the highest tidal volume we tested (Figure 6), such tolerance error would be 105 mL, a value far higher than the one we found (26 mL) using the worst K_1_ – K_2_ combination (Figure 6). Moreover, from a clinical perspective the errors we found in determining typical tidal volumes ( ≤ 4%) are very low. For instance, when addressing protective mechanical ventilation, the typical tidal volume range of 6–8 mL per kg of body weight (28) gives a window of ≈30% amplitude. Moreover, when separating patient phenotypes by low and high respiratory compliance (derived from volume measurement), the intermediate phenotype (29) ranges (40–50 mL/cmH_2_O), i.e., a 20% amplitude window again much greater than our potential errors in volume measurement ( ≤ 4%; Figure 6). Whereas, this work was carried out on the assumption of adult mechanical ventilation (flow and volume ranges), the procedure can be adapted to monitoring infant ventilation by adequately scaling the sizes of the pneumotachograph and the resuscitation bag and the ranges of flows and volumes.

## Conclusions

This work provides solid laboratory evidence that accurate pneumotachographs can be very easily constructed, and that they can be calibrated with no need for conventional and expensive gold standard reference devices in advanced laboratories. Accordingly, the proposed approach facilitates the low-cost and simple availability of pneumotachographs for accurately controlling mechanical ventilation in low-resource settings, either by incorporating them into the ventilators or as external measuring devices for quality control.

## Data availability statement

The original contributions presented in the study are included in the article/Supplementary material, further inquiries can be directed to the corresponding author/s.

## Author contributions

RF conceived the study, analyzed the data, wrote the manuscript, and supervised the work. MR-L carried out the experiments. GT, DN, DG, and JO contributed to discussing the research, interpreting the data, and providing insightful comments. All authors contributed to the article and approved the submitted version.

## Conflict of interest

The authors declare that the research was conducted in the absence of any commercial or financial relationships that could be construed as a potential conflict of interest.

## Publisher's note

All claims expressed in this article are solely those of the authors and do not necessarily represent those of their affiliated organizations, or those of the publisher, the editors and the reviewers. Any product that may be evaluated in this article, or claim that may be made by its manufacturer, is not guaranteed or endorsed by the publisher.

## References

[1] Truog RD Mitchell C Daley GQ. The toughest triage - allocating ventilators in a pandemic. N Engl J Med. (2020) 382:1973–5. 10.1056/NEJMp2005689 32202721

[2] Murthy S Leligdowicz A Adhikari NKJ. Intensive care unit capacity in low-income countries: a systematic review. PLoS ONE. (2015) 10:e0116949. 10.1371/journal.pone.0116949 25617837 PMC4305307

[3] Farré R Trias G Solana G Ginovart G Gozal D Navajas D. Novel approach for providing pediatric continuous positive airway pressure devices in low-income, under resourced regions. Am J Respir Crit Care Med. (2019) 199:118–20. 10.1164/rccm.201808-1452LE 30265582 PMC6353013

[4] Garmendia O Rodríguez-Lazaro MA Otero J Phan P Stoyanova A Dinh-Xuan AT. Low-cost, easy-to-build noninvasive pressure support ventilator for under-resourced regions: open source hardware description, performance and feasibility testing. Eur Respir J. (2020) 55:2000846. 10.1183/13993003.00846-2020 32312862 PMC7173672

[5] Pearce JM. A review of open source ventilators for COVID-19 and future pandemics. F1000Res. (2020) 9:218. 10.12688/f1000research.22942.2 32411358 PMC7195895

[6] Howie SR Hill SE Peel D Sanneh M Njie M Hill PC. Beyond good intentions: lessons on equipment donation from an African hospital. Bull World Health Organ. (2008) 86:52–6. 10.2471/BLT.07.042994 18235890 PMC2647344

[7] Badnjevic A Gurbeta L Jimenez ER Iadanza E. Testing of mechanical ventilators and infant incubators in healthcare institutions. Technol Health Care. (2017) 25:237–50. 10.3233/THC-161269 28387686

[8] Petrucci N De Feo C. Lung protective ventilation strategy for the acute respiratory distress syndrome. Cochrane Database Syst Rev. (2013) 2013:CD003844. 10.1002/14651858.CD003844.pub4 23450544 PMC6517299

[9] Curley GF Laffey JG Zhang H Slutsky AS. Biotrauma and ventilator-induced lung injury: clinical implications. Chest. (2016) 150:1109–17. 10.1016/j.chest.2016.07.019 27477213

[10] Brochard L Slutsky A Pesenti A. Mechanical ventilation to minimize progression of lung injury in acute respiratory failure. Am J Respir Crit Care Med. (2017) 195:438–42. 10.1164/rccm.201605-1081CP 27626833

[11] Govoni L Dellaca' RL Peñuelas O Bellani G Artigas A Ferrer M.. Actual performance of mechanical ventilators in ICU: a multicentric quality control study. Med Devices. (2012) 5:111–9. 10.2147/MDER.S35864 23293543 PMC3534536

[12] Lyazidi A Thille AW Carteaux G Galia F Brochard L Richard JC. Bench test evaluation of volume delivered by modern ICU ventilators during volume-controlled ventilation. Intens Care Med. (2010) 36:2074–80. 10.1007/s00134-010-2044-9 20862452

[13] Schena E Massaroni C Saccomandi P Cecchini S. Flow measurement in mechanical ventilation: a review. Med Eng Phys. (2015) 37:257–64. 10.1016/j.medengphy.2015.01.010 25659299

[14] Yeh MP Gardner RM Adams TD Yanowitz FG. Computerized determination of pneumotachometer characteristics using a calibrated syringe. J Appl Physiol Respir Environ Exerc Physiol. (1982) 53:280–5. 10.1152/jappl.1982.53.1.280 7118642

[15] Cross TJ Kelley EF Hardy TA Isautier JMJ Johnson BD. The syringe potentiometer: a low-cost device for pneumotachograph calibration. J Appl Physiol (1985). (2019) 127:1150–62. 10.1152/japplphysiol.00150.2019 31487222 PMC6850981

[16] Rohrer F. Der Strömungswiderstand in den menschlichen Atemwegen und der Einfluss der unregelmässigen Verzweigung des Bronchialsystems auf den Atmungsverlauf in verschiedenen Lungenbezirken. Pflüger's Arch. (1915) 162:225–99. 10.1007/BF01681259

[17] Smierciew K Butrymowicz D Karwacki J Gagan J. Numerical prediction of homogeneity of gas flow through perforated plates. Processes. (2021) 9:1770. 10.3390/pr9101770

[18] Whitaker S. The Forchheimer equation: a theoretical development. Transp Porous Med. (1996) 25:27–61. 10.1007/BF00141261

[19] Huang K Wan JW Chen CX He LQ Mei WB Zhang MY. Experimental investigation on water flow in cubic arrays of spheres. J Hydrol. (2013) 492:61–8. 10.1016/j.jhydrol.2013.03.039

[20] Spaeth J Steinmann D Kaltofen H Guttmann J Schumann S. The pressure drop across the endotracheal tube in mechanically ventilated pediatric patients. Paediatr Anaesth. (2015) 25:413–20. 10.1111/pan.12595 25491944

[21] Jarreau PH Louis B Dassieu G Desfrere L Blanchard PW Moriette G. Estimation of inspiratory pressure drop in neonatal and pediatric endotracheal tubes. J Appl Physiol (1985). (1999) 87:36–46. 10.1152/jappl.1999.87.1.36 10409556

[22] Flevari AG Maniatis N Kremiotis TE Siempos I Betrosian AP Roussos C. Rohrer's constant, K2, as a factor of determining inspiratory resistance of common adult endotracheal tubes. Anaesth Intensive Care. (2011) 39:410–7. 10.1177/0310057X1103900311 21675060

[23] Alsalaet J Munahi BS Al-Sabur R Al-Saad M Ali AK Shari BA. Laminar flowmeter for mechanical ventilator: Manufacturing challenge of Covid-19 pandemic. Flow Meas Instrum. (2021) 82:102058. 10.1016/j.flowmeasinst.2021.102058 34611384 PMC8484002

[24] Weight WD (ed),. Manual of Applied Field Hydrogeology. Appendix B: Relationship of Water Density Viscosity to Temperature. 1st ed. New York, NY: McGraw-Hill Education (2001). Available online at: https://www.accessengineeringlibrary.com/content/book/9780070696396 (accessed July 20, 2022).

[25] Prezioso D Di Domenico D Pane M Ciccarelli D D'Errico G. Ion specificity in determining physico-chemical properties of drinking water. Food Sci Technol Campinas. (2019) 39:485–90. 10.1590/fst.34717

[26] Tang Y Turner MJ Yem JS Baker AB. Calibration of pneumotachographs using a calibrated syringe. J Appl Physiol (1985). (2003) 95:571–6. 10.1152/japplphysiol.00196.2003 12704091

[27] Medicine Health Regulatory Agency (UK). Rapidly Manufactured Ventilator System. (2020). Available online at: https://assets.publishing.service.gov.uk/government/uploads/system/uploads/attachment_data/file/879382/RMVS001_v4.pdf (accessed July 20, 2022).

[28] Ogbu OC Martin GS Murphy DJ. A few milliliters of prevention: lung-protective ventilation decreases pulmonary complications. Crit Care Med. (2015) 43:2263–4. 10.1097/CCM.0000000000001234 26376257 PMC4576716

[29] Panwar R Madotto F Laffey JG van Haren FMP. Compliance phenotypes in early acute respiratory distress syndrome before the COVID-19 pandemic. Am J Respir Crit Care Med. (2020) 202:1244–52. 10.1164/rccm.202005-2046OC 32805143 PMC7605177

