# Supplementary material for: Simple low-cost construction and calibration of accurate pneumotachographs for monitoring mechanical ventilation in low-resource settings
Source: Front Med (Lausanne). 2022 Aug 1;9:938949. doi: 10.3389/fmed.2022.938949 (PMC9376320; doi:10.3389/fmed.2022.938949)
Supplement: Supplementary file 1 [file Data_Sheet_1.PDF]

SUPPLEMENTARY MATERIAL

Simple low-cost construction and calibration of accurate pneumotachographs for monitoring mechanical ventilation in low-resource settings

Ramon Farré[1,2,3,*], Miguel A. Rodríguez-Lázaro[1], David Gozal[4], Gerard Trias[5], Gorka Solana[6], Daniel Navajas[1,2,7], Jorge Otero[1,2]

[1]Unitat de Biofísica i Bioenginyeria, Facultat de Medicina i Ciències de la Salut, Universitat de Barcelona, Barcelona, Spain; [2]CIBER de Enfermedades Respiratorias, Madrid, Spain; [3]Institut Investigacions Biomèdiques August Pi Sunyer, Barcelona, Spain; [4]Department of Child Health, The University of Missouri School of Medicine, Columbia, MO, USA; [5]Department d'Infrastructures i Enginyeria Biomedica, Hospital Clínic, Barcelona, Spain; [6]Faculdade de Engenharias e Tecnologias, Universidade Save, Maxixe, Mozambique; [7]Institute for Bioengineering of Catalonia (IBEC), Barcelona Institute of Science and Technology, Barcelona, Spain.

*Corresponding author:

Prof. Ramon Farré
Unitat de Biofísica i Bioenginyeria
Facultat de Medicina i Ciències de la Salut
Casanova 143
08036 Barcelona, Spain
Email: rfarre@ub.edu

CHARACTERIZATION OF THE PRESSURE-FLOW RELATIONSHIP IN THE PNEUMOTACHOGRAPHS

The values of K_1 and K_2 corresponding to the five pneumotachographs that were constructed were measured by connecting them to the outlet of a high-accuracy computerized syringe designed for pneumotachograph calibration (Pulmonary Waveform Generator (PWG), MH Custom Design & Mfg LC. Utah, USA). The pressure signals (P) measured across the pneumotachograph by a low-pass filtered (8-pole Butterworth, 32 Hz) differential transducer (±2 cmH_2O, LCVR; Celesco, Canoga Park, CA, USA) were compared with the corresponding constant flows (V') generated by the gold standard syringe. Fitting the Rohrer model to the P-V' data from each pneumotachograph provided the corresponding values of K_1 and K_2 for both flow directions (syringe inhalation and exhalation).

CALIBRATION OF A NON-LINEAR PNEUMOTACHOGRAPH FROM TWO MANEUVERS OF KNOWN VOLUME

Theoretical background.

For positive flows, the general pressure-flow relationship described by the Rohrer model ($P = K_1 \cdot V' + K_2 \cdot |V'| \cdot V'$) is simplified to $P = K_1 \cdot V' + K_2 \cdot V'^2$, which is a quadratic equation on variable V':

$$K_2 \cdot V'^2 + K_1 \cdot V' - P = 0 \qquad (1)$$

that can be solved as

$$V' = \alpha \cdot [\sqrt{(1+\beta \cdot P)} - 1] \qquad (2)$$

where coefficients α and β are

$$\alpha = \frac{K_1}{2 \cdot K_2} \qquad \beta = \frac{4 \cdot K_2}{K_1^2} \qquad (3)$$

The air volume corresponding to a maneuver involving a flow waveform V'(t) is

$$V(t) = \int V^{'}(t)\cdot dt \qquad (4)$$

In case the flow signal is sampled with a time interval ΔT (sampling frequency = 1/ΔT), Equation 4 becomes

$$V = \sum V^{'}_{i} \cdot \Delta T \qquad (5)$$

where subindex i denotes the flow samples, and summation ∑ extends for the total number of data samples from the maneuver.

Should the flow signal be measured by the pressure drop (P) across a resistor characterized by K_1 and K_2, Eq. 2 can be used in Eq. 5 to compute the air volume (V) in the maneuver as

$$V = \Delta T \cdot \sum \alpha\cdot[\sqrt{(1+\beta\cdot P_i)} - 1] \qquad (6)$$

Characterization of a Rohrer-type pneumotachograph (i.e. to determine K_1 and K_2) can be achieved from the pressure (P_1 and P_2) recordings from two maneuvers with known volumes V_1 and V_2, respectively. Indeed, according to Eq. 6,

$$V_1 = \sum V^{'}_{1}\cdot\Delta T = \Delta T \cdot \alpha \cdot \sum [\sqrt{(1+\beta\cdot P_{1i})} - 1] \qquad (7)$$

$$V_2 = \sum V^{'}_{2}\cdot\Delta T = \Delta T \cdot \alpha \cdot \sum [\sqrt{(1+\beta\cdot P_{2i})} - 1] \qquad (8)$$

The system of Equations 7 and 8 can be simply reduced to the following equation having only one unknown (β):

$$V_1 \cdot \sum\left[\sqrt{(1+\beta\cdot P_{2i})} - 1\right] - V_2 \cdot \sum\left[\sqrt{(1+\beta\cdot P_{1i})} - 1\right] = 0 \qquad (9)$$

which can be solved for β by minimizing ε in

$$\varepsilon = V_1 \cdot \sum\left[\sqrt{(1+\beta\cdot P_{2i})} - 1\right] - V_2 \cdot \sum\left[\sqrt{(1+\beta\cdot P_{1i})} - 1\right] \qquad (10)$$

and, subsequently solved for α by using either Eq. 7 or 8. Once α and β are determined, the values of K_1 and K_2 characterizing the pneumotachograph can be computed from Eq. 3 as

$$K_1=\frac{2}{\alpha\cdot\beta} \qquad K_2=\frac{\beta\cdot K_1^2}{4} \qquad (11)$$

Practical implementation.

Minimization of ε from P_{2i}, P_{1i} and V_1 and V_2 (Eq. 10) can be carried out by any conventional algorithm. However, given that this minimization is a converging process, we used a simple method based on starting with a reasonable initial value of β (e.g. β derived from values of K_1 and K_2 in Table 1, main publication) and subsequently computing ε and adjusting β until getting minimization (usually after ≈ 10 steps).

SIMPLE ACCURATE MEASUREMENT OF AIR VOLUME BY WATER DISPLACEMENT

Theoretical background.

The two-maneuver-based method described above for calibrating a non-linear pneumotachograph requires accurately knowing the air volume of the maneuvers. Usually, in a respiratory lab, this could be achieved either by generating the maneuver with a reference syringe or by using a high-accuracy reference pneumotachograph to measure the volume of the maneuver. However, such gold standard devices are not easily available in low-resource settings. Accordingly, we have devised a simple, low-cost procedure for accurately measuring the air volume in each maneuver requiring no volume/flow gold standard devices.

The proposed setting, which is based on measuring the volume of water displaced by the airflow maneuver, which is collected by an external recipient and measured (Figure 2, main publication). The procedure is based on assuming that when a volume V_2 of room air is introduced into a container with fully compliant walls having an air volume V_1 and a water liquid phase, V_1 and V_2 are mixed at constant room pressure (P) and temperature (T). Figure S1.A shows the case when air volume V_1 is introduced to the chamber by means of a fully compliant bag-in-box setting: regardless of the relative humidities of V_1 and V_2, the final volume of the gas mixture (V_{12}) is $V_1 + V_2$. However, in case a bag-in-box setting is not used (Figure S1.B), the volume of the air mixture may be different from V_1+V_2 depending on the relative humidities of the air (H). Indeed, whereas the number of mols of dry gas (those excluding water vapor) in the mixture will be the addition of the ones from V_1 and V_2, the number of mols of water vapor would depend on initial and final air humidities. The number of dry-gas mols (n_1 and n_2) of V_1 and V_2, each with initial relative humidities H_1 and H_2, respectively, are:

$$n_1 = \frac{V_1 \cdot (P - P_{H2O} \cdot H_1)}{R \cdot T} \qquad n_2 = \frac{V_2 \cdot (P - P_{H2O} \cdot H_2)}{R \cdot T} \qquad (12)$$

where P_{H2O} is the water vapor pressure at T, and R is the constant of gases. Hence, the volume of the air mixture (V_{12}) will be the one corresponding to the number of mols of dry air ($n_1 + n_2$) at humidity H_{12}:

$$V_{12} = \frac{V_1 \cdot (P - P_{H2O} \cdot H_1) + V_2 \cdot (P - P_{H2O} \cdot H_2)}{(P - P_{H2O} \cdot H_{12})} \qquad (13)$$

Thus, the volume of the air mixture (V_{12}) depends on initial (H_1, H_2) and final (H_{12}) humidities. Whereas the air mixture would finally get an equilibrium state with the water liquid phase, i.e. $H_{12} = 1$ (100% water vapor saturation), H_{12} may exhibit other values during the transient phase towards equilibrium depending on the condensation/evaporation rates in the system.

According to Eq. 13, the increase in volume ($\Delta V = V_{12} - V_1$) experienced by the gas mixture in the chamber −which would be simply V_2 in case condensation/evaporation of water vapor was not involved− is

$$\Delta V = V_1 \cdot \left[\frac{(P - P_{H2O} \cdot H_1)}{(P - P_{H2O} \cdot H_{12})} - 1\right] + V_2 \cdot \frac{(P - P_{H2O} \cdot H_2)}{(P - P_{H2O} \cdot H_{12})} \qquad (14)$$

In the trivial case where initial and final relative humidities are 100% (all gases saturated with water vapor ($H_1 = H_2 = H_{12} = 1$), ΔV would be equal to V_2. However, in case the air introduced in the chamber is not saturated with water vapor ($H_2<1$), as is common in ambient air, ΔV would depend on whether V_1 was initially saturated and on whether the mixture achieves final saturation.

If the process is observed for a time period much shorter than the time required for eventual equilibrium by evaporation/condensation of water in the whole setup, the initial mols of water vapor would be maintained whatever the initial conditions of relative humidity and therefore $\Delta V = V_2$. By contrast, if the process is observed on a time scale long enough for the air mixture to achieve humidity equilibrium with liquid water (i.e., $H_{12} = 1$), two possible situations can be considered:

a) If V_1 was already saturated ($H_1 = 1$), Eq. 14 results in

$$\Delta V_a = V_2 \cdot \frac{(P - P_{H2O} \cdot H_2)}{(P - P_{H2O})} \qquad (15)$$

indicating that ΔV_a ($\geq V_2$ for $H_2<1$) is the change of volume experienced when V_2 increases humidity from H_2 to $H_2=1$ (100% saturation).

b) If V_1 was initially at the same humidity conditions as V_2, (i.e. $H_1=H_2$) -for instance when the air chamber is initially replaced with room air-, then Eq. 14 results in

$$\Delta V_b = V_1 \cdot \left[\frac{(P - P_{H2O} \cdot H_2)}{(P - P_{H2O})} - 1\right] + V_2 \cdot \frac{(P - P_{H2O} \cdot H_2)}{(P - P_{H2O})} \qquad (16)$$

indicating that both V_1 and V_2 increase the content of water vapor when changing from humidity $H_1 = H_2$ to 100% saturation. Accordingly, $\Delta V_b > \Delta V_a$ and whereas ΔV_a only depends on the air volume introduced into the chamber (V_2), ΔV_b depends on both V_2 and the initial air volume in the chamber (V_1).

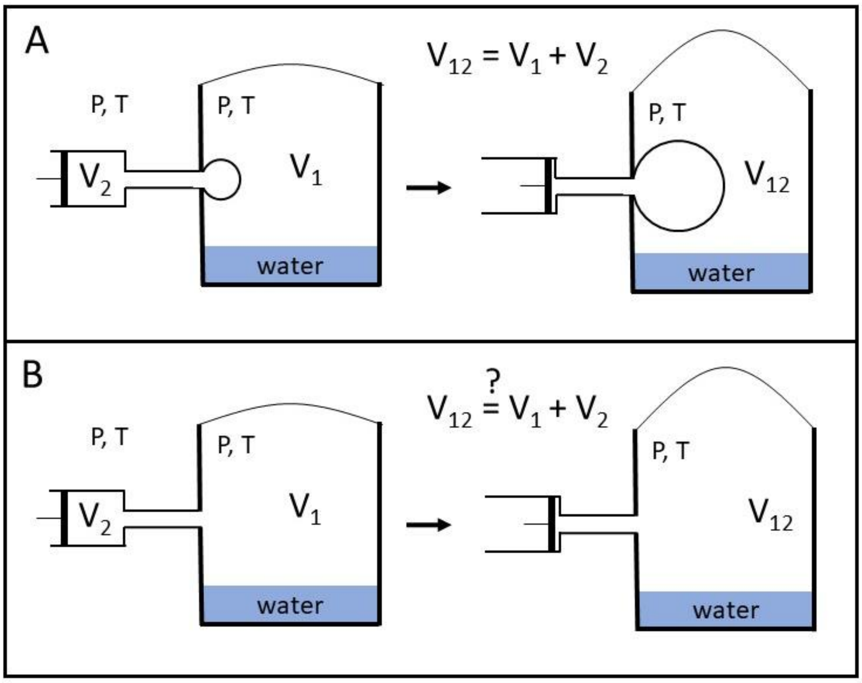

Figure S.1. Volume of a gas mixture. (A) Setting with a fully compliant bag-in-box. (B) No bag-in-box setting. A volume V_1 of air is inside a chamber containing liquid water, and a volume V_2 of room air is introduced into the chamber. The top wall of the chamber is fully compliant so that pressure (P) inside the chamber is kept constant and equal to ambient pressure. Temperature is constant along the process. In (A) the volume of the air mixture (V_{12}) = $V_1 + V_2$. In (B), V_{12} may be different from $V_1 + V_2$, depending on the initial and final relative humidities of air. See text for explanation.

It is interesting to note that a minor correction of the measured water volume is required to account for the compliance of the setting (Figure 2, main publication). Indeed, at the end of the maneuver, the setting remains compressed at a pressure (ΔP) corresponding to a column of water of height h (Figure 2.A, main publication), hence ΔP

$= \rho \cdot g \cdot h$ ($g = 9.8$ m/s^2). Accordingly, the volume of displaced water measured does not exactly correspond to the volume of air introduced into the chamber by the syringe since a minor fraction is shunted by the compression of the air within the syringe, and by the compliance of the chamber walls if not perfectly rigid (e.g., in the case of a plastic bottle chamber). Therefore, the volume of water that is displaced and measured (V_w) is the volume indicated by the syringe piston position (V_p) minus the volume shunted ($V_{sh} = \Delta P \cdot C_{sh}$) by the total compliance of the setting (C_{sh}), and hence $V_p = V_w + \rho \cdot g \cdot h \cdot C_{sh}$. The magnitude of such required correction, which depends on the specific implementation of the system in Figure 2 (main publication), is reduced as h and/or C_{sh} are reduced. To carry out the correction in our setting, C_{sh} was measured by occluding the chamber outlets and by recording the pressure increases caused by small air volumes injected with a conventional syringe.

Practical implementation

The volume of displaced water was assessed by weighting it with a low-cost electronic kitchen scale and using water density ($\rho = 1$ g/mL). Remarkably, this scale was compared with a high-accuracy laboratory scale (range 0−1000 g, 0.01g; WLC 1/A2/C2, Radwag, Radom, Poland), exhibiting accuracy within ±2 g. For the specific setting we employed (Figure 2, main publication) this correction was small since, in the setting, h was only 5.1 cm for a measured volume of displaced water (V_w) of 1 L. Taking into account that the measured compliance of the setting (C_{sh}) was 3.77 mL/cmH_2O (corresponding to a pressure increase of 5.3 cmH_2O when 20 mL of air were injected into the occluded system), the magnitude of correction for $V_w = 1$ L would be V_{sh} ($= \Delta P \cdot C_{sh}$) would be 19.2 mL (i.e. a 1.9% correction in volume).

INSPIRATORY FLOW WAVEFORMS FOR TESTING THE PNEUMOTACHOGRAPH DURING MECHANICAL VENTILATION

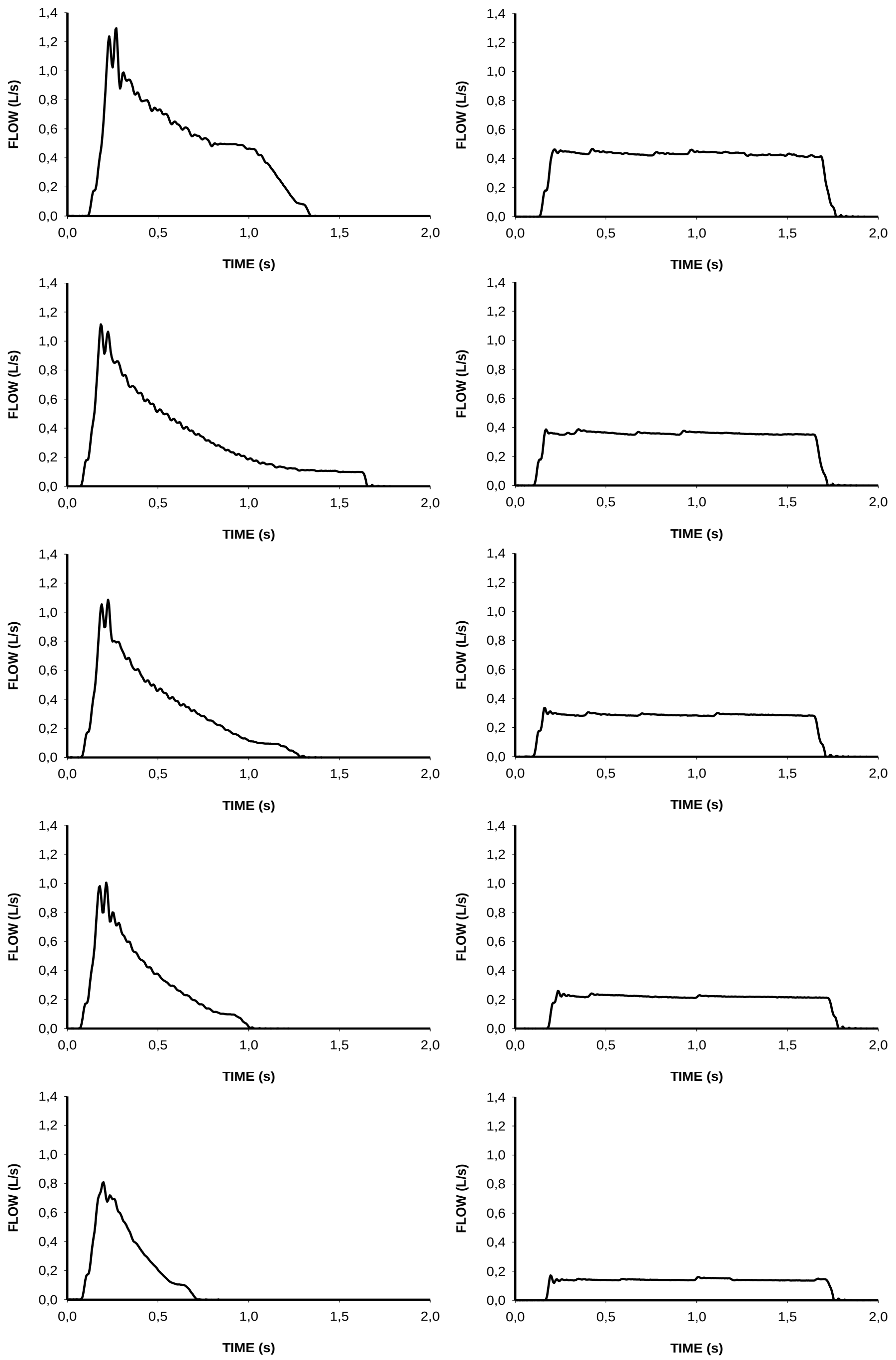

Figure S.2. Inspiratory flow when testing the low-cost pneumotachograph during pressure-controlled (left) and volume-controlled (right) mechanical ventilation as described in the Methods subsection “Assessment of the low-cost pneumotachograph and calibration procedure to monitor mechanical ventilation” of the main publication.
